# Trends in the utilization of antihypertensive medications among Palestine refugees in Jordan, 2008-2012

**DOI:** 10.1186/s40545-015-0036-4

**Published:** 2015-05-18

**Authors:** Rawan Saadeh, Dima Qato, Ali Khader, Yousef Shahin, Akihiro Seita

**Affiliations:** Health Department, United Nations Relief and Works Agency Headquarters, Bayader Wadi Seer, 11814, Amman Jordan; Department of Pharmacy Systems, Outcomes and Policy, University of Illinois at Chicago, 833 South Wood Street, Chicago, Illinois USA

**Keywords:** Essential medicines, Refugees, Palestine, Antihypertensives

## Abstract

**Objectives:**

The purpose of this study is to describe trends in the utilization of antihypertensive medications, overall and by type of medication, specifically thiazide diuretics, as well as uncontrolled hypertension, in the Palestine refugee population in Jordan between 2008 and 2012.

**Methods:**

We analyzed aggregate procurement data on antihypertensive medications derived from the United Nations Relief and Works Agency (UNRWA) pharmacy records between 2008 and 2012. Antihypertensive medications were aggregated and utilization was calculated overall and for specific types of antihypertensive medications (e.g. *β*-blockers, diuretics). We used the WHO (World Health Organization) defined daily dose (DDD) methodology, often used to evaluate drug utilization patterns using aggregate data, to calculate utilization defined as DDDs per 100 persons with hypertension. In addition, UNRWA medical records were used to measure the prevalence of uncontrolled hypertension in the primary care setting. Uncontrolled hypertension was defined as a systolic/diastolic blood pressure ≥140/90 in at least 2 out of 3 readings, one of which is the most recent reading, during the year for a patient diagnosed with hypertension.

**Results:**

Overall, total utilization of antihypertensive medications has not changed between 2008 and 2012; hypertensive patients persistently used at least 2 antihypertensive medications daily (range 200–280 DDDs/100 patients with hypertension) during this five-year period. However, there is significant variation in utilization patterns by type of antihypertensive medication. While Angiotensin Converting Enzyme Inhibitors (ACE-I) were persistently the most commonly used antihypertensive medication, there utilization significantly (P < 0.05) declined by 26%. However, there was a statistically significant increase of 124% in the utilization of thiazide diuretics. Further, the prevalence of uncontrolled hypertension has also declined at a rate of 3% annually between 2008 and 2012.

**Conclusion:**

Our findings indicate that the STGs for hypertension management implemented in 2009 as part of UNRWA's essential drug program have increased the utilization of thiazide diuretics, and potentially contributed to improvements in hypertension control. This study also demonstrates that feasibility of drug utilization studies in monitoring and evaluating trends in the use of essential medications in low-resource settings.

## Introduction

Hypertension is a persistent and leading risk factor for cardiovascular morbidity and mortality globally [1], including countries in the Middle East for which there is a substantial, yet often overlooked, population of Palestine refugees [2]. As of 2012, there were more than 5 million Palestine refugees living in Jordan, Lebanon, Syria and Palestine (West Bank and Gaza) with more than 15% of the adult population suffering from hypertension, 30% of which are considered high risk [3]. Since 1951, the United Nations Relief and Works Agency (UNRWA) has been responsible for the delivery of primary health care (PHC) services at no cost, including the provision of essential medicines such as antihypertensive medications, for this chronically vulnerable refugee population [3,4]. Despite the critical role of antihypertensive medications in the management of hypertension, and in the prevention of complications associated with uncontrolled hypertension, there is currently no information on their utilization in the Palestine refugee population.

Understanding the utilization of antihypertensive medications among Palestine refugees is particularly important for several reasons. First, hypertension is increasingly prevalent in an aging refugee population, [3] and information on whether patterns in the utilization of antihypertensive medications align with treatment guidelines is imperative for ongoing efforts to improve hypertension management. This is particularly important considering UNRWA’s 2009 implementation of evidence-based standard treatment guidelines (STGs) which relied on the hypertension treatment recommendations of the Joint National Commission-VII (JNC-7) [5] that have since been updated to JNC-VIII [6]. The impact of these STGs on the utilization of antihypertensive medications, however, is not known. Second, over the last several years UNRWA spending on antihypertensive medications has increased with annual expenditures totaling more than $1.8 million in 2012 [7]. Thus, understanding whether trends and patterns of antihypertensive medication utilization not only align with STGs, but are also effective in improving hypertension control is critical in reducing complications and costs associated with uncontrolled hypertension. Finally, ensuring the essential medicines program, anchored in human rights principles, [8,9] effectively promotes better health in an under-recognized and often ignored population of Palestine refugees is a humanitarian imperative.

In order to better characterize patterns of antihypertensive medication use in the Palestine refugee population, we leverage existing data derived from UNRWA pharmacy procurement records for Jordan to describe trends in the utilization of antihypertensive medications between 2008 and 2012, and discuss the potential impact of STG implementation on utilization patterns as hypertension control We focus on Jordan because it has more than 2 million UNRWA Palestine refugees, which, aside from Palestine, is the largest population of Palestine refugees in the Middle East [4].

## Methods

We used existing aggregate procurement data derived from the UNRWA pharmacy records to examine the utilization of antihypertensive medications between 2008 and 2012 in Jordan. UNRWA procurement data captures detailed information on quantities, strength and dosage forms of medications distributed to patients that utilize UNRWA PHCs in Jordan. These data are not directly linked to patient records.

We also retrieved de-identified information on blood pressure readings from the UNRWA PHC health management information system (HMIS) for patients with a diagnosis of hypertension, We used this information to describe the annual prevalence of uncontrolled hypertension among the Palestine refugee patient population treated by general practitioners at the UNRWA PHCs in Jordan between 2008 and 2012 . Uncontrolled hypertension was defined as a systolic/diastolic blood pressure ≥140/90 in 2 out of 3 readings, one of which is the most recent reading, during the treatment year.

Antihypertensive medications were classified based on the World Health Organization (WHO) Anatomic Therapeutic Chemical (ATC) system which are depicted in Table 1 [10].

**Table 1:** **Changes in the utilization (DDDs per 100 hypertensive patients per year) of antihypertensive medications among the Palestine refugee hypertensive patient population in Jordan between 2008 (pre-STG) and 2012 (post-STG)**

**ATC codes**	**Therapeutic class**	**Antihypertensive utilization, % of total**		**Change in utilization**	**Percent change in utilization**
		**2008**	**2012**	**2008-2012**	**2008-2012**
	Total**	280.5 (100%)	275.3(100%)	−5.23	−1.86%
C02AB01	Alpha-adrenergic agonists	2.22 (0.8%)	2.55 (0.9%)	+0.33	+14.86%
C09AA02	ACE inhibitors**	178.1 (63.5%)	132.5 (48.1%)	−45.67	−25.64%
C09CA01	Angiotensin II receptor antagonists**	0 (0%)	7.7 (2.8%)	+7.71	-
C07AA05, C07AB03	Beta blockers	53.9 (19.2%)	55.4 (20.1%)	+1.43	+2.65%
C08CA05, C08DB01, C08CA01	Calcium channel blockers	23.8 (8.5%)	26.97 (9.8%)	+3.2	+13.46%
C03AA03,C03AX01, C03CA01,C03DB01	Diuretics**	22.4 (8.0%)	50.2 (18.2%)	+27.77	+123.9% (only more than 100% is significant)

DDD = defined daily dose, ACE = Angiotensin-converting enzyme; Standard Treatment Guidelines (STG) for hypertension management implemented in 2009 at UNRWA primary health clinics (PHCs).

**The binomial proportion 95% confidence interval excludes the null value of zero difference (P-value <0.05).

We also used the WHO defined daily dose (DDD) methodology, which is often used to evaluate drug utilization using aggregate data, to calculate utilization of antihypertensive medications using our procurement data. We obtained the DDD values from the WHO ATC/DDD index to derive DDDs for each medication. DDD is a measure that represents that average daily maintenance dose for the main indication of a drug in adults. It is used to aggregate data on different doses, strengths, and formulations to enable comparisons of utilization across different medications, different populations and over time. For example, 10 DDDs/100 patients per day for a medication or class of medications indicates that 10% of the population on average receive a certain medication or group of medications daily. In our study, utilization is defined as number of DDDs per 100 persons with hypertension (patient population) per day. Our hypertension sample was based on the number of patients over the age of 40 diagnosed with hypertension at UNRWA PHCs between 2008 and 2012 (Table 2).

**Table 2:** **Prevalence of Hypertension among the Palestine Refugee Population ≥ 40 years in Jordan, 2008–2012**

**Year**	**Total registered population**	Total served population* (% of total registered population)	**Served population ≥ 40 years**	**Number of patients ≥ 40 years with a Diagnosis of Hypertension**	**Prevalence of hypertension among patients ≥ 40 years**
2008	1,951,603	1104607 (56.6%)	307,935	42,495	13.80%
2009	1,983,733	1110890 (56.0%)	312,953	46,317	14.80%
2010	1,999,485	1050035 (52.5%)	324,743	49,361	15.20%
2011	2,047,367	1074251(52.5%)	323,423	52,718	16.30%
2012	2,110,114	1175021(55.7%)	367,434	53,278	14.50%

*Number of Palestine refugees that utilize UNRWA Primary Health Care (PHC) services.

The number of DDDs for all antihypertensive medications were aggregated and utilization was calculated for antihypertensive medications overall, by antihypertensive therapeutic category (e.g. β-blockers, diuretics) and for individual antihypertensive medications (e.g. enalapril). With the exception of amiloride/hydrocholorothiazide combination product procured in 2012, all medication products were single-ingredient products. We describe the prevalence of antihypertensive utilization measured as DDD/100 persons with hypertension between 2008 and 2012.

**Figure 1:**
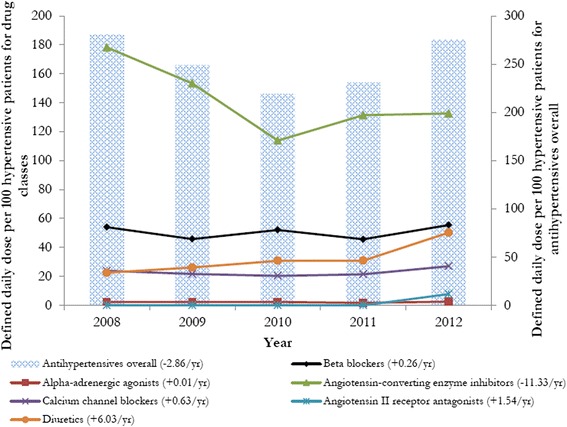
Trends in the defined daily dose (DDD) of antihypertensives overall and by type of drug classes per 100 patients from 2008 to 2012. The trend (change in DDD per 100 patients) was calculated using simple linear regression model. T-tests for change in DDD per 100 patients across years was not statistically significant for antihypertensives overall [P = 0.787].T-tests for change in DDD per 100 patients across years was statistically significant for diuretics [P = 0.044]. F-test for differences in trends between the drug classes was statistically significant [P = 0.009].

**Figure 2:**
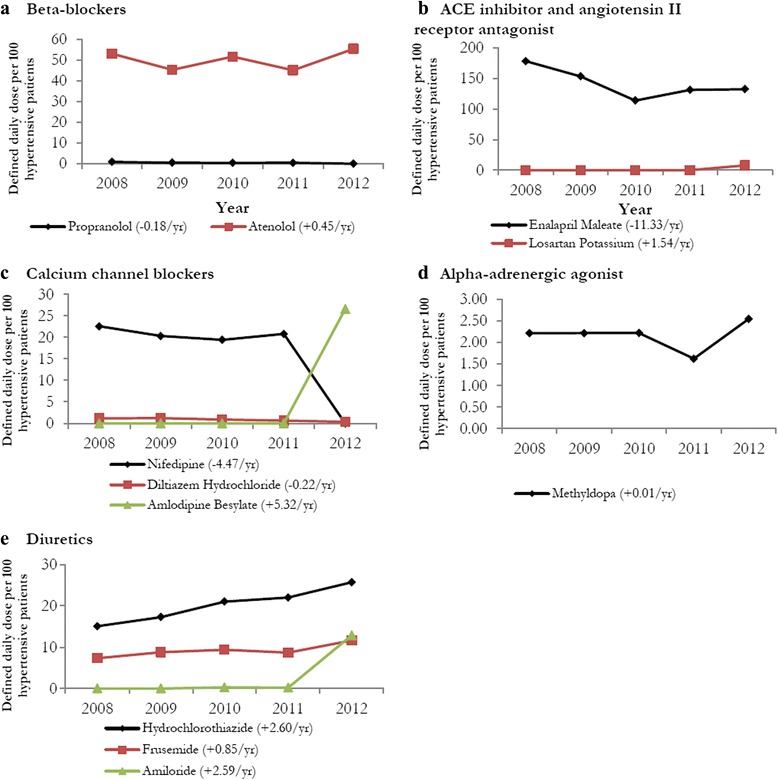
Trends in the defined daily dose (DDD) of antihypertensive drugs per 100 patients from 2008 to 2012. **a-e** The trend (change in DDD per 100 patients) was calculated using simple linear regression model. T-tests for change in DDD per 100 patients across years was statistically significant for propranolol [P = 0.033], diltiazem hydrochloride [P = 0.009] and hydrochlorothiazide [P = 0.001].

We used simple linear regression models to calculate the trend (change in DDD per 100 hypertensive patients). We also used the t-test to determine the statistical significance of the trend (change in DDD over time) between all therapeutic categories (Figure 1) and between individual types of medications (Figure 2). A statistically significant change in the utilization of antihypertensive medications overall and by therapeutic class between 2008 and 2012 was also examined based on the exclusion of zero difference in the 95% binomial confidence interval. F-test was conducted to assess differences in trends between the drug classes. All analyses were conducted in SAS statistical software. Patient information was de-identified prior to analyses. This study was approved by the University of Illinois Institutional Review Board that is responsible for the ethics of research conducting related to human subjects. Written informed consent was obtained from the patient for the publication of this report and any accompanying images

## Results

Table 2 provides a summary of the refugee population served by UNRWA PHCs and the prevalence of hypertension between 2008 and 2012. In 2012, more than 1 million people utilized the UNRWA PHCs with approximately 400,000 aged ≥40 years. According to these data derived from UNRWA medical records, the number of refugees with hypertension in Jordan has increased between 2008 and 2012. However, the prevalence of hypertension has not changed substantially between 2008 and 2012. In 2012, 53,278 patients or 14.5% of the served population aged ≥ 40 years was diagnosed with hypertension (defined as systolic/diastolic blood pressure ≥ 140/90).

Figure 1 depicts trends in the utilization (DDDs per 100 hypertension patients per day or DDDs) for antihypertensive medications overall and by therapeutic class for the UNRWA refugee hypertensive patient population. Overall, hypertensive patients used at least 2 antihypertensive medications daily (or 2 DDDs per patient with hypertension) during this five-year period. The average decline (−2.86 DDDs/year) for all hypertensive medications was not statistically significant (p-value = 0.787). However, there was a statistically significant difference in utilization over time between antihypertensive therapeutic classes. For example, the only significant trend or annual change over time was for the utilization of diuretics (+6.03 DDDs; p < 0.05). While there was a notable decline in the utilization of ACE-I (−11.3/year), the trend was not statistically significant.

Table 1 summarizes changes in utilization of antihypertensive medication by ATC code and therapeutic category between 2008 (base year) and 2012. The utilization of antihypertensive medications slightly declined from 280.5 to 275.3 DDDs between 2008 and 2012 (+5.23 DDDs [95% CI 0.87-9.59]). The utilization of ACE-I declined by 25% from 178.14 to 132.47 DDDs (−45.7 DDDs [95% CI 35.91-55.43]). However, ACE-I persistently accounted for the majority of antihypertensive medication utilization; 64% and 48% of total DDDs from antihypertensive medications in 2008 and 2012, respectively, were for ACE-I. Further, angiotensin II receptor antagonist were not utilized between 2008 and 2011, but accounted for 7.7 DDDs in 2012.

The most substantial increase in utilization was observed for diuretics (123.9%). Diuretics significantly (P-value < 0.05) increased from 22.4 DDDs to 50.2 DDDs per 100 hypertensive patients (+27.8 DDD [95% CI 18.99-36.55]) in 2008 and 2012, respectively. Our results do not indicate a statistically significant change in trends in the utilization of other classes of antihypertensive medications.

Figure 2 depicts trends in the utilization of specific antihypertensive medications by therapeutic class. Among β-blockers, the utilization of propranolol was consistently lower than atenolol during the 5-year time period. Although the use of atenolol increased from 53.1 to 55.41 DDDs per 100 hypertensive patients (+0.45 DDDs/year) during this observation period, the increase was not significant. The decline (−0.90 DDDs per 100 hypertensive patients) in the utilization of propranolol was also not significant during this period (P = 0.34).

Among ACE-I and angiotensin II receptor antagonists, enalapril’s utilization declined from 178.14 to 132.47 DDDs per 100 hypertensive patients (−11.33 DDDs/year) between 2008 and 2012. This decline (−45.7 DDDs per 100 hypertensive patients) was the largest among all antihypertensive medications. Losartan potassium was only used among the UNRWA patients in Jordan during the year 2012 (7.71 DDDs per 100 hypertensive patients).

Among calcium channel blockers, the utilization of nifedipine and diltiazem hydrochloride declined by 22.6 and 0.81 DDDs per 100 hypertensive patients, respectively. The change in the utilization of diltiazem hydrochloride was not significant (P = 0.519). Nifedipine’s utilization was higher than diltiazem hydrochloride between 2008 and 2011. In 2012, no utilization was observed for nifedipine, while amlopidine was utilized for the first time. Methyldopa had the smallest increase in utilization (+0.33 DDDs per 100 hypertensive patients) among all antihypertensive drugs between 2008 and 2012.

All three diuretic medications had an increase in utilization between 2008 and 2012. Amiloride, available since 2010 to the UNRWA PHC patients, had the highest increase in utilization (+12.86 DDDs per 100 hypertensive patients) among the diuretics during this period. However, hydrochlorothiazide was consistently the most commonly used diuretic and increased from 15.1 to 25.3 DDDs per 100 hypertensive patients during this period (P = 0.062). The increase in the utilization (+4.27 DDDs per 100 hypertensive patients) of furosemide was the lowest among diuretics during the 5-year time period.

**Figure 3:**
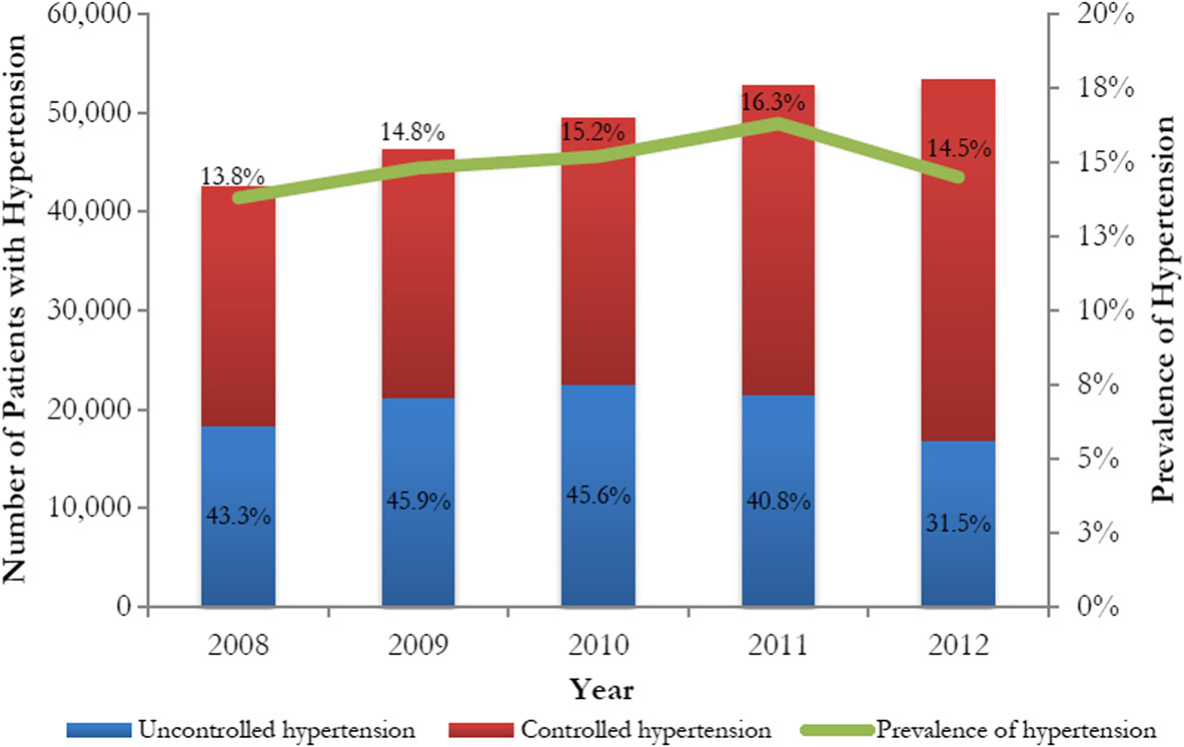
Prevalence of uncontrolled hypertension among the Palestine refugee population (>40 years old) from 2008 to 2012. The trend (change in prevalence of hypertension) was calculated using simple linear regression model. T-tests for change in prevalence of any and uncontrolled hypertension across years were not statistically significant [P = 0.396 and P = 0.130, respectively]. The average increase in the prevalence of any hypertension per year was 0.29%. The average decrease in the percentage of hypertensive patients with uncontrolled high blood pressure per year was 2.87%.

Figure 3 illustrates the patterns in the prevalence of hypertension as well as uncontrolled hypertension in the UNRWA patient population for which these antihypertensive medications were procured. Despite an increasing prevalence of hypertension among patients aged 40 years and older, the number of patients with uncontrolled hypertension has significantly declined from 43.3% or 18,400 patients to 31.5% or 16,783 patients. The decline in the prevalence of uncontrolled hypertension by 11.80% was statistically significant (P < 0.001). The chi-square test for the difference in prevalence of uncontrolled hypertension between 2008 and 2012 was found to be statistically significant [P < 0.0001].

## Discussion

To our knowledge, this is the first study to examine trends in the utilization of medications in the Palestine refugee patient population. We used readily available procurement data derived from UNRWA pharmacy records to examine trends in the utilization of antihypertensive medications among Palestine refugees in Jordan between 2008 and 2012. Our findings indicate that the total utilization of antihypertensive medications has not changed substantially over time. However, trends in the types of antihypertensive medications used and the prevalence of uncontrolled hypertension has significantly improved among the Palestine refugee patient population. This initial drug utilization study increases our understanding of the effectiveness of UNRWA’s essential medicines program, particularly the STG’s, in improving cardiovascular health, particularly hypertension, in Palestine refugees.

Palestine refugees with hypertension were persistently using, on average, between two and three antihypertensive medications daily, suggesting combination therapy regimens were common. Angiotensin Converting Enzyme Inhibitors (ACE-I) were the most commonly used antihypertensive medication and accounted for the majority of antihypertensive medications throughout the 5-year period. However, ACE-I utilization declined in 2012 and was being replaced by diuretics, which may have important clinical implications in the prevention of complications in hypertension patients with co-morbid conditions such as diabetes, such as renal impairment, and warrants further investigation.

Between 2008 and 2012, the use of diuretics increased more than two-fold, particularly hydrochlorothiazide. The utilization of all other types of antihypertensive medications did not change. These findings are not surprising considering the JNC-VII hypertension treatment guidelines that recommend diuretics, specifically thiazide diuretics, as first-line agents and suggest the STGs updated in 2009 were an effective strategy in improving prescribing, and consequently, utilization of antihypertensive medications among the UNRWA patient population. However, the thiazide diuretics are not always indicated in the management of hypertension, for example in diabetic patients, and further investigation of these utilization patterns in the patient population would better elucidate the appropriateness of the patterns we observed in this study.

While the utilization of β-blockers did not change between 2008 and 2012, it was persistently greater than the utilization of diuretics. However, the difference lessened over time with increasing thiazide diuretic utilization. These changing patterns of antihypertensive utilization are important considering the updated JNC-VIII [6] hypertension treatment guidelines that recommend β-blockers only as later line alternative therapies. Therefore the current UNRWA STGs should be revised to better align with the JNC-VIII guidelines (vs. JNC-VII) that discourage the use of β-blockers that have been associated with a differential risk for cardiovascular events [11].

Trends in the prevalence of uncontrolled hypertension were also improving during the same 5-year period. The gradual decline in the prevalence of uncontrolled hypertension among the patient population to 31% is notable, and may be associated with the increasing use of thiazide diuretics in combination with ACE-I which are the recommended first-line therapies in the current JNC-VIII guidelines [6]. Our findings suggest that programming efforts to improve the appropriate use of essential medicines in the Palestine refugee population have potentially important implications on health outcomes such as lower incidence of cardiovascular complications (e.g. myocardial infarction, heart failure, and stroke) often associated with uncontrolled hypertension [12]. Future investigations should include patient-level analyses that better examine the impact of appropriate use of antihypertensive medications across various cardiovascular risk profiles, and their associated outcomes, in Palestine refugee.

Improvement in both the utilization of antihypertensive medications, and uncontrolled hypertension, is particularly noteworthy in comparison to findings for the general population in Jordan. For example, a study conducted in 2009 examined antihypertensive medication use for 416 men and women patients aged 18–94, undergoing hypertension management at primary health care clinics in Jordan. This study found combination therapy with β-blockers and diuretics to be the most frequently used antihypertensive therapeutic regimen [13]. While JNC-VII guidelines had recommended diuretics or β-blockers as first line therapies, their combined use is suboptimal as evident in the high prevalence (more than 50%) of uncontrolled hypertension (SBP/DBP ≥140/90) in the patient population. These findings further suggest that implementation of STGs are often critical in promoting the appropriate use of medicines in the management of NCD such as hypertension [14].

Finally, our study demonstrates the feasibility of conducting drug utilization studies in refugee settings using procurement data derived from existing pharmacy records. Our findings further suggest that drug utilization studies can be used as a tool to monitor and evaluate the effectiveness of essential medicines programs, such as adherence to standard treatment guidelines in primary health care. Future studies should examine the utilization of medications for other common and costly non communicable diseases (NCDs) such as diabetes, hypercholesterolemia, and chronic obstructive pulmonary disease (COPD).

## Limitations

Our study has several limitations. The use of aggregate procurement data may not adequately represent utilization or appropriateness of treatment at the individual level. For example, we are unable to discern differences in the types of medications use as mono- or combination therapy at the patient-level. Therefore we are unable to specifically identify what combinations were used, and whether and how they affect hypertension control at the patient level. Our data on both the utilization of antihypertensive medications and the prevalence of uncontrolled hypertension represents patients undergoing treatment at UNRWA PHC and may not be generalizable to the general Palestine refugee population. Therefore, future studies should investigate the use of medicines, and specific health outcomes measures, in the community setting.

## Conclusions

While trends in the total utilization of antihypertensive medications have not changed between 2008 and 2012, the types of antihypertensive medications used and the prevalence of uncontrolled hypertension has significantly improved among Palestine refugees in Jordan. Our findings indicate that the STGs for hypertension management implemented in 2009 as part of UNRWA’s essential drug program have not only increased the utilization of thiazide diuretics, which better aligns with international treatment guidelines for hypertension, but have potentially contributed to improvements in hypertension control. Future research to examine the patterns in the utilization antihypertensive medications, and hypertension control, at the community-level are warranted.
